# Experiences with cytoreduction surgery plus hyperthermic intraperitoneal chemotherapy in Taiwan

**DOI:** 10.1097/MD.0000000000007306

**Published:** 2017-06-30

**Authors:** Mao-Chih Hsieh, Chang-Yun Lu, Wei-Wen Chang, Szu-Yuan Wu, Ping-Kun Hsiao, Tse-Jia Liu

**Affiliations:** a Division of General Surgery, Department of Surgery, Wan Fang Hospital; b Division of General Surgery, Department of Surgery, School of Medicine, College of Medicine; c Department of Radiation Oncology, Wan Fang Hospital; d Department of Internal Medicine, School of Medicine, College of Medicine, Taipei Medical University; e Institute of Toxicology, College of Medicine, National Taiwan University, Taipei; f Department of Biotechnology, Hungkuang University, Taichung, Taiwan.

**Keywords:** completeness of cytoreduction score, cytoreduction surgery, early postoperative intraperitoneal chemotherapy (EPIC), hyperthermic intraperitoneal chemotherapy (HIPEC), peritoneal cancer index (PCI), peritoneal carcinomatosis, peritonectomy

## Abstract

Our hospital was the first institution to offer cytoreduction surgery (CRS) plus hyperthermic intraperitoneal chemotherapy (HIPEC) in Taiwan. Therefore, we report our experience and outcomes among patients who underwent HIPEC.

Since 2002, 164 eligible patients underwent HIPEC, and we excluded cases of laparoscopic or prophylactic HIPEC. The cases were categorized according to whether they were treated before 2012 (Period 1: 80 cases) or after 2012 (Period 2: 84 cases).

The rates of surgical morbidity were 46.3% during Period 1 and 20.2% during Period 2 (*P* < .01), and the rates of severe complications were 25% during Period 1 and 9.5% during Period 2 (*P* < .01). The 5-year overall survival rate was 35.8%, with rates of 13.4% for gastric cancer, 27.3% for colon cancer, 70.0% for appendiceal cancer, and 52.4% for ovarian cancer (median follow-up: 34 months). The survival rate was 42.1% when we achieved a cytoreduction score of 0/1, compared with 21.1% in the group with a cytoreduction score of 2/3 (*P* < .01). Severe complications were associated with a 5-year survival rate of 23.4%, compared with 37.9% among cases without severe complications (*P* = .01). Complete cytoreduction was achieved in 78.6% of the patients if they underwent their first surgery at our hospital.

We have become an experienced hospital for CRS plus HIPEC. Although our complication rate for CRS plus HIPEC was high, it was within the acceptable range. Long-term survival was achieved in a few cases.

## 1 Introduction

Intraperitoneal carcinomatosis that is caused by intra-abdominal malignancy usually leads to intestinal obstruction with very poor outcomes. Systemic chemotherapy only yields limited results, although some patients have been managed with curative intent after the introduction of cytoreduction surgery (CRS) and hyperthermic intraperitoneal chemotherapy (HIPEC).^[1,2]^ Although this treatment has not been well accepted by most surgeons and oncologists, because of its high rates of complications and mortality, we continue to perform CRS plus HIPEC using improving CRS techniques, including peritonectomy procedures. Since 2000, the Wan-Fang Hospital has been the only institution to offer CRS plus HIPEC to patients with peritoneal surface malignancies in Taiwan. In this report, we describe our experience and outcomes among patients who underwent HIPEC since 2002.

## 2 Materials and methods

This study included consecutive cases of CRS plus HIPEC that were performed from 2002 until December 2014 at our institution. We excluded patients who underwent laparoscopic or prophylactic HIPEC so that we can exclusively analyze the results of CRS plus HIPEC. All patients had peritoneal carcinomatosis that was caused by gastric cancer, colorectal cancer, appendiceal cancer (pseudomyxoma peritonei), ovarian cancer, peritoneal mesothelioma, ruptured hepatoma, or other malignancies. CRS was performed in accordance with the techniques that have been described by Sugarbaker^[1]^ and Yonemura.^[2]^ This treatment was approved by our institutional ethics review board (F911003), and all the patients had provided their informed consent for the treatment after receiving explanations of cytoreduction surgery, peritonectomy, HIPEC, and the potential postoperative complications.

Before surgery, all the patients underwent chest radiography, abdominal and pelvic computed tomography, a barium enema, routine blood tests, and hepatic/renal/pulmonary/cardiac function tests. Total parenteral nutritional (TPN) support was prescribed for 5 to 7 days before the surgery for patients with poor oral intake and poor nutritional status.

After entering the peritoneal cavity, we recorded the preoperative peritoneal cancer index (PCI).^[3]^ After the cytoreduction, the completeness of cytoreduction score (CS-0 to CS-3) was recorded according to the Sugarbaker classification.^[4]^ We performed HIPEC immediately after the CRS using the coliseum technique and a roller-pump heat-exchanger perfusion machine. The HIPEC procedure was started before any intestinal anastomosis and/or before closure of the abdomen. All the HIPEC procedures were performed while maintaining an intraperitoneal temperature of 42°C to 43°C for 60 minutes, and the timing was only started after the intraperitoneal temperature reached 42°C. The postoperative PCI was also recorded. The chemotherapeutic agents were selected based on the type of cancer, and included mitomycin C, cisplatin, etoposide, epirubicin, and docetaxel. For example, we used mitomycin C, cisplatin, and etoposide for gastric cancer, although we changed the treatment to docetaxel in 2008. For appendiceal cancer and colon cancer, we used mitomycin C in a standard regimen.

Early postoperative intraperitoneal chemotherapy (EPIC) was performed for some patients, who started the 5-day treatment on the first postoperative day. On each day, the chemotherapeutic agent was kept within the peritoneal cavity for 23 hours, which was followed by 1 hour of drainage.

Complications were recorded according to the Clavien-Dindo System (grade 1: mild complications, grade 5: death).^[5]^ Operative mortality was defined as any death within 30 days of surgery or during the same hospitalization.

Patients were closely followed using laboratory testing and imaging every 3 months for the first 3 years and then every 6 months thereafter. Recurrence was most often diagnosed using radiographic findings and/or by tissue biopsy findings in select circumstances. The patients were evaluated by an oncology group and selectively treated using adjuvant systemic therapy.

All the procedures were performed by the first author, although the second author joined the team after 2012. For the present study, the cases were categorized according to whether they underwent surgery before 2012 (n = 80) or after 2012 (n = 84). Continuous data were presented as mean ± standard deviation and were compared using Student *t* test. Categorical data were presented as number (%) and were compared using the *χ*^2^ test or Fisher exact test, as appropriate. Survival outcomes were analyzed for all HIPEC cases and according to disease, cytoreduction score, and/or the presence of severe complications. Survival outcomes were calculated by using the Kaplan–Meier method and log rank (Mantel–Cox) model. A *P* value of <.05 was considered statistically significant. All the statistical analyses were performed using SPSS software (SPSS Inc, Chicago, IL).

## 3 Results

**Figure 1:**
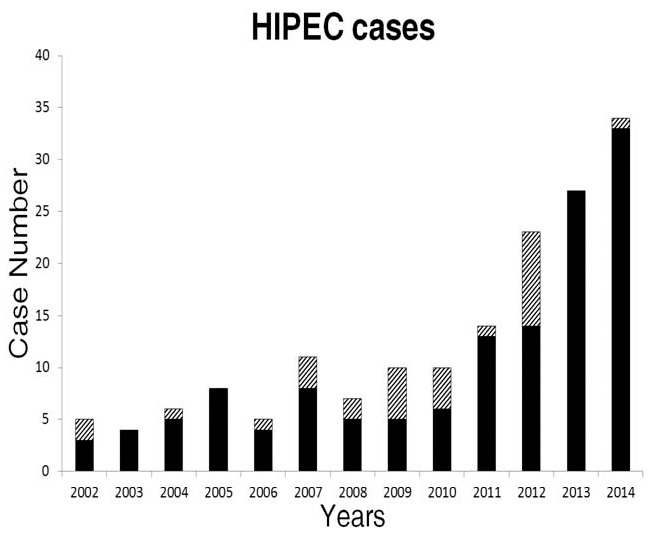
Cases receiving hyperthermic intraperitoneal chemotherapy according to year. The diagonal shading indicates referrals from other hospitals.

In recent years, increasing numbers of patients have visited our hospital for CRS plus HIPEC (Fig. 1). A total of 198 procedures were performed for 164 patients who were included in our analyses. The patients included 70 men and 94 women, who had an average age of 52.5 years (range: 22–83 years). Their primary diseases included gastric cancer (54 cases, 32.9%), appendiceal cancer (38 cases, 23.2%), colon cancer (32 cases, 19.5%), ovarian cancer (17 cases, 10.4%), and others (e.g., ruptured hepatoma, mesothelioma, liposarcoma, and primary peritoneal malignancy; total: 23 cases, 13.9%). Only 29 cases (17.7%) were referred from other hospitals/physicians, with 19 cases being treated during Period 1 (23.8%) and 10 cases being treated during Period 2 (11.9%, *P* = .05). Ninety-four patients (57.3%) underwent prior surgery for the original malignancy, with 38 prior surgeries (47.5%) during Period 1 and 56 prior surgeries (66.7%) during Period 2 (*P* = .01). The prior surgeries included gastrectomy (n = 12), colectomy (n = 33), appendectomy (n = 46), oophorectomy (n = 31), cholecystectomy (n = 7), hepatectomy (n = 8), splenectomy (n = 1), small bowel resection (n = 6), debulking (n = 20), and open-and-close cases (n = 13). More appendectomies were observed during Period 2 (30 cases, 35.7%), compared with during Period 1 (16 cases, 20%, *P* = .03). A total of 112 patients received TPN, with 58 patients (35.4%) having previously received chemotherapy/targeted therapy for the original malignancy (Table 1).

**Table 1:**
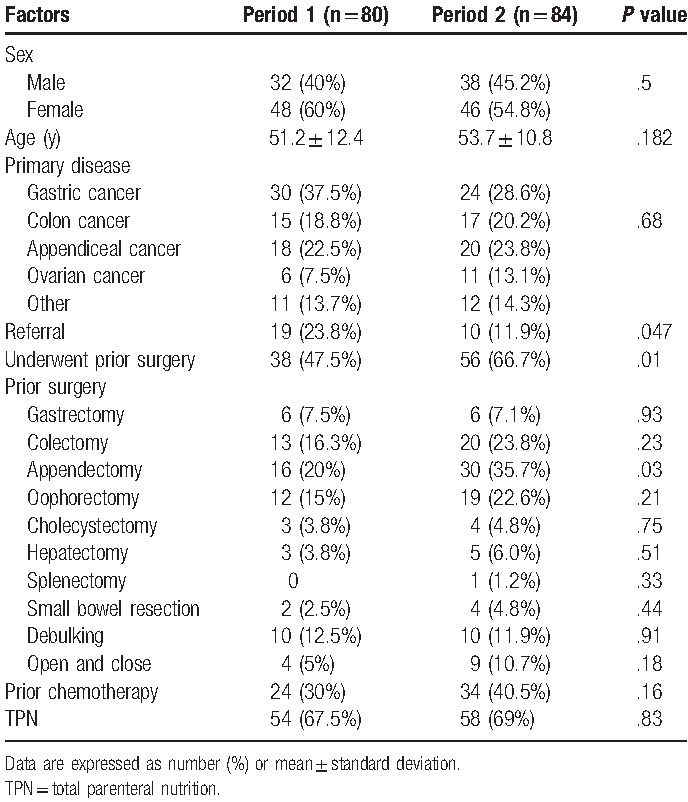
Demographic data from each period.

### 3.1 Preoperative and postoperative management

The mean preoperative PCI was 15.9 ± 11.7 during Period 1 and 20.0 ± 13.3 during Period 2 (*P* = .1). Cytoreduction was performed using gastrectomy (n = 49), colectomy (n = 82), cholecystectomy (n = 81), splenectomy (n = 26), oophorectomy (n = 51), hepatectomy (n = 11), small bowel resection (n = 61), diaphragm resection (n = 10), peritonectomy (n = 96), and colostomy (n = 31). More splenectomies were performed during Period 2 (18 cases, 21.4%), compared with during Period 1 (8 cases, 10%, *P* = .05). In addition, more peritonectomies were performed during Period 2 (56 cases, 66.7%), compared with during Period 1 (40 cases, 50%, *P* = .03).

EPIC was administered in 88 cases (53.7%), and postoperative chemotherapy was administered to complete the treatment course in 99 cases (60.4%) (Table 2). The postoperative PCI was lower during Period 1 (5.5 ± 10.0), compared with during Period 2 (9.0 ± 13.2, *P* = .06).

**Table 2:**
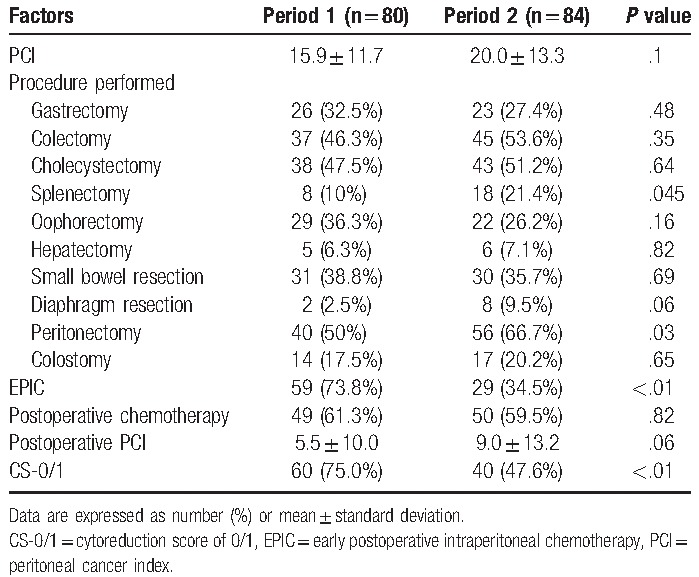
Management during and after the operation.

### 3.2 Surgical outcomes

The surgical morbidity rate was 46.3% during Period 1 and 20.2% during Period 2 (*P* < .01) (Table 3). The morbidities were related to the surgical procedures in 31.3% of these cases during Period 1 and in 13.1% of these cases during Period 2 (*P* < .01). Severe complications (grade 3–5) were observed in 20 cases during Period 1 (25%) and in 8 cases during Period 2 (9.5%, *P* < .01).

**Table 3:**
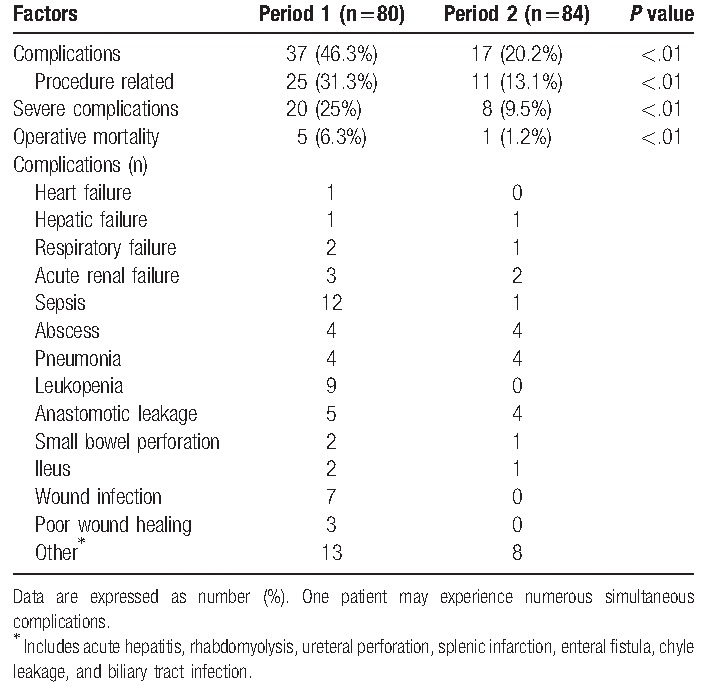
Grade 3 to 4 complications among patients who underwent cytoreduction surgery plus hyperthermic intraperitoneal chemotherapy.

### 3.3 Survival outcomes

**Figure 2:**
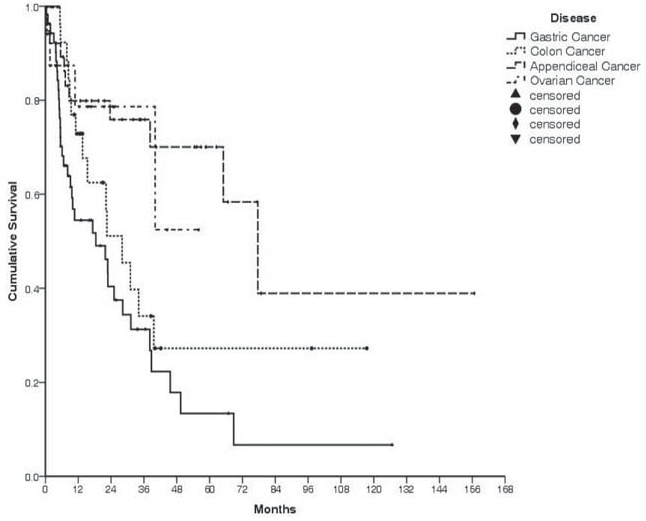
Survival according to disease. Gastric cancer: 13.4%, colon cancer: 27.3%, appendiceal cancer: 70.0%, and ovarian cancer: 52.4%.

**Figure 3:**
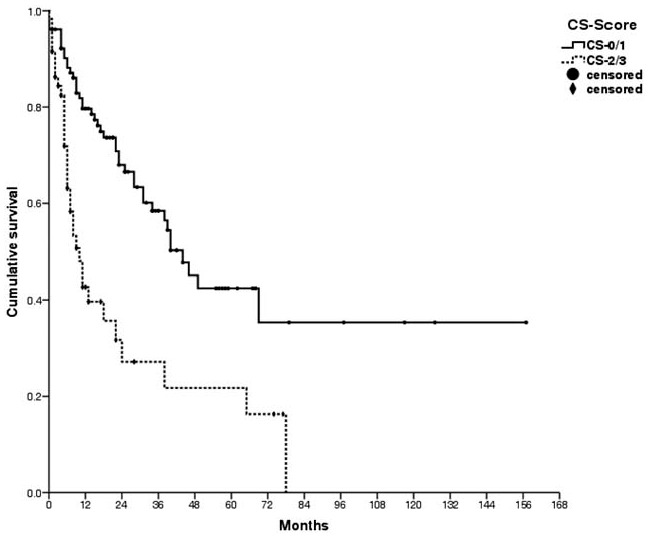
Survival was improved by more complete cytoreduction (42.1% for scores of 0/1 vs 21.1% for scores of 2/3, *P* < .01).

**Figure 4:**
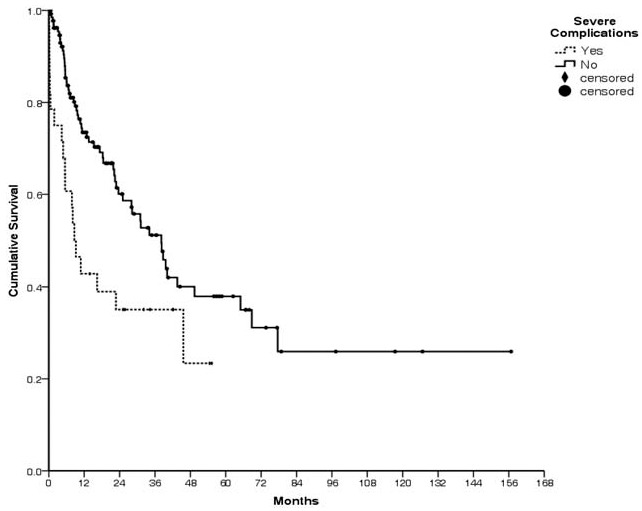
A better survival rate (37.9%) was observed in the absence of severe complications. Survival was poor in cases with severe complications (23.4%, *P* = .01).

The 5-year overall survival rate for all patients was 35.8% (median follow-up: 34 months), with rates of 13.4% for gastric cancer, 27.3% for colon cancer, 70.0% for appendiceal cancer, and 52.4% for ovarian cancer (Fig. 2). The median survival times were 18.4 months for gastric cancer, 28.0 months for colon cancer, and 77.6 months for appendiceal cancer. The survival rate was 42.1% when CS-0/1 cytoreduction was achieved, compared with only 21.1% in the CS-2/3 group (*P* < .01, Fig. 3). Severe complications were associated with a 5-year survival rate of 23.4%, compared with 37.9% for patients with no severe complications (*P* = .01, Fig. 4).

The overall survival rates in Period 2 were 72.4% at 1 year, 58.8% at 2 years, and 58.8% at 3 years. In Period 1, the overall survival rates were 64.5% at 1 year, 52.9% at 2 years, and 45.2% at 3 years. Although the survival outcomes were better in Period 2, compared with Period 1, the difference was not statistically significant, which may be related to the short follow-up time in Period 2.

## 4 Discussion

HIPEC is not a well-known procedure in Taiwan, even among surgeons and oncologists. However, increasing numbers of patients have visited us to undergo HIPEC during recent years. Interestingly, although data have been published that support CRS plus HIPEC treatment for peritoneal carcinomatosis, the number of referred cases at our institution has not increased. In 1994, the first author began administering HIPEC therapy at the Veterans General Hospital-Taipei, where he treated 27 patients.^[6]^ The first author subsequently began administering HIPEC at Wan-Fang Hospital in 2000, and has achieved good results (mainly in treating gastric cancer). Since that time, numerous patients have personally sought HIPEC, rather than being referred from physicians at other hospitals. Unfortunately, some patients (especially during Period 2) received HIPEC after third-line chemotherapy, and it was very difficult to achieve positive outcomes in those cases, as the patients had very late-stage disease.

If multiple large metastatic lesions (>2.5 mm) were found during surgery at the mesentery or serosa of the whole small intestines (regions 9–12 based on the PCI definition, from the upper jejunum to the lower ileum), CRS was abandoned because it could not remove all of the lesions. Furthermore, resection of a large amount of the small intestine will cause a short bowel condition. In addition, HIPEC was terminated immediately when we detected a poor cardiac response or poor urine output during surgery, and we excluded patients with these conditions from this study.

Gastric cancer is the fifth most common cause of cancer-related deaths in Taiwan,^[7]^ and a large proportion of our cases involved gastric cancer. Unfortunately, the prognosis for gastric cancer was the poorest in the present study, and the 5-year overall survival for patients with this disease was only 13.4% (1-year: 54.5%, 2-year: 40.4%, and 3-year: 31.2%). Similarly, Yang et al^[8]^ have used CRS plus HIPEC to treat gastric cancer with peritoneal carcinomatosis, and they reported 1 and 2-year survival rates of 50% and 42.8%, respectively. Furthermore, Glehen et al^[9]^ have reported a 5-year overall survival rate of 13%, and a rate of 23% when complete cytoreduction was achieved, which are similar to our findings. Yonemura et al^[10]^ have reported a 5-year survival rate of 6.7%, with rates of 13% for complete cytoreduction and 2% for incomplete cytoreduction.

In the present study, patients with colon cancer had 1, 2, and 3-year survival rates of 72.9%, 51.1%, and 34.1%, respectively (median survival: 28 months). Similarly, Verwaal et al^[11]^ have reported 1, 3, and 5-year survival rates of 75%, 28%, and 19%, respectively, for patients with colon cancer. Haslinger et al^[12]^ have reported a 5-year survival rate of 38.2% with a median survival time of 45.2 months.

Moran et al^[13]^ have reported a 5-year survival rate of 84% after complete resection for patients with appendiceal cancer. In the present study, the 5-year survival rate for patients with appendiceal cancer was 70.0%, regardless of the cytoreduction score. However, there were only 17 patients with ovarian cancer in our data, and the median survival cannot currently be calculated. Nevertheless, our results are comparable to the reported data from many other countries, which are summarized in Table 4.

**Table 4:**
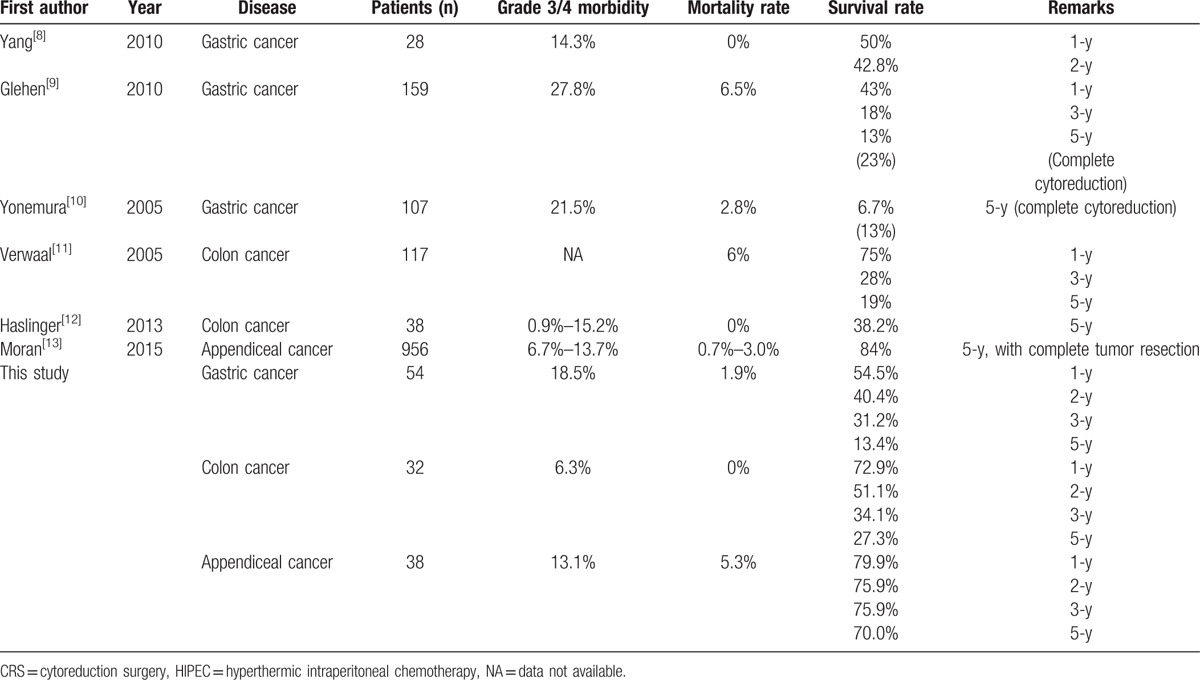
Summary of CRS and HIPEC outcomes for treating diseases with peritoneal carcinomatosis.

CRS plus HIPEC is associated with high rates of morbidity and mortality, and this is a major treatment concern. For example, gastric cancer is associated with a morbidity rate of 27.8% and a mortality rate of 6.5%,^[9]^ and the reported grade 3/4 morbidity and mortality rates are 12% to 71.2% and 0.9% to 5.6%, respectively.^[14,15]^ Furthermore, 1 systematic review^[16]^ reported that the grade 3/4 morbidity rate was 12% to 52% and the mortality rate was 0.9% to 5.8%. We also found that our overall morbidity rate was 32.9%, although it decreased from 46.3% during Period 1 to 20.2% during Period 2. Similarly, the procedure-related complication rate decreased from 31.3% during Period 1 to 13.1% during Period 2. Thus, it appears that our complication rates decreased noticeably after 2012.

Numerous reports have discussed the necessary surgical experienced that is needed to decrease morbidity and mortality rates. For example, Yan et al^[17]^ suggested that the minimum experience was 70 cases, although Smeenk et al^[15]^ suggested 130 procedures were necessary (based on the percentage of complete cytoreduction) and Polanco et al^[18]^ suggested 180 procedures were necessary. Moreover, a multicenter study revealed that 8 of 33 centers and 6 of 47 surgeons achieved a significant reduction in early oncological failure for patients with pseudomyxoma peritonei after a median of 100 procedures (range: 78–284) and 96 procedures (range: 86–284), respectively.^[19]^ Moran et al^[13]^ also found that 20 years of experience provided a reduction in the rates of grade 3/4 morbidity (from 13.7% to 6.7%) and mortality (from 3.0% to 0.7%). In the present study, 80 patients were treated before 2012 and >100 patients were personally treated by the first author. Thus, our growing experience with CRS plus HIPEC may explain the decrease in our morbidity rate after 2012, as we only observed 7 cases of operative mortality (4.3%) and one death during Period 2. These results suggest that we are now an experienced hospital for CRS plus HIPEC.

Achieving scores of CS-0/1 was a major prognostic factor in the present study. However, Period 1 involved a higher proportion of CS-0/1 cases (vs Period 2), as more complicated cases were treated during Period 2, with significant increases in the Period 2 values for mean PCI and postoperative PCI. In addition, fewer patients had undergone prior surgery during Period 1. Interestingly, when patients underwent the first CRS at our institution, complete cytoreduction was achieved in 78.6% of the cases, although complete cytoreduction was only achieved in 52.1% of the cases that underwent debulking at other hospitals. In addition, patients with gastric cancer or colon cancer frequently underwent debulking at other hospitals, which might have contributed to our difficulty in achieving CS-0/1. Furthermore, it was difficult to achieve a CS-0/1 cytoreduction in many patients in Period 2. For example, tumors that were >2.5 cm and adhered to a vital structure (e.g., inferior vena cava) might have resulted in a tumor fragment that was densely adhered to the vessel. By classification, this is CS-3 cytoreduction, although we avoided disastrous complications using subsequent multimodality treatment that included focally intense radiotherapy. As a result, the survival rates for these cases improved during Period 2. Thus, we continued performing CRS plus HIPEC for these patients, despite the increasing proportion of CS-2/3 in Period 2.

There are three limitations in this study. First, we have only encountered a limited number of cases, despite Wan-Fang Hospital being a tertiary referral hospital. This might be related to patients personally seeking out CRS plus HIPEC, rather than being referred for treatment, which might limit the number of cases, as most patients with peritoneal carcinomatosis are not aware of CRS plus HIPEC therapy. Second, patients who came to us had usually experienced many operations and/or chemotherapies, which made it difficult to achieve complete cytoreduction. Fortunately, we have learned from these diverse cases, and have been able to decrease our morbidity and mortality rates. Third, we used a retrospective design and were unable to compare our results with patients who only received conventional systemic chemotherapy. Thus, a randomized controlled trial may be needed to validate our findings, which would be supported by the increasing numbers of cases during recent years.

## 5 Conclusion

In conclusion, our rate of complications after CRS plus HIPEC was high at our center, although it was within the acceptable range based on results from other studies. Long-term survival was achieved in select cases, and our results were encouraging for cases of gastric, colon, and appendiceal cancers. Moreover, our achievement of CS-0/1 scores and less severe complications were likely associated with the positive patient outcomes that we observed. Therefore, we believe that we have become an experienced hospital for CRS plus HIPEC, despite being the only institution in Taiwan to offer it for treating peritoneal surface malignancies. However, additional cases involving different diseases should be examined to further improve our experience and patient outcomes.

## Acknowledgments

We acknowledge the statistical support of the Biostatistics Center, College of Management, Taipei Medical University, Taipei, Taiwan. We thank Ms Hui-Ching Hsu for contacting the patients and Ms Hui-Chun Hou for assistance with the data registration. We also thank Editage (www.editage.com) for English language editing.
